# Challenges in adapting a stroke unit in a middle-income country: warning about costs and underfunding to achieve the Brazilian Ministry of Health’s benchmark

**DOI:** 10.3389/fpubh.2024.1264292

**Published:** 2024-02-01

**Authors:** Natalia Cristina Ferreira, Gustavo José Luvizutto, Silméia Garcia Zanati Bazan, Luana Aparecida Miranda Bonome, Fernanda Cristina Winckler, Daniel Fabiano Barbosa dos Santos, Cristiane Lara Mendes Chiloff, Gabriel Pinheiro Modolo, Carlos Clayton Macedo de Freitas, Pasqual Barretti, Marcos Christiano Lange, Marcos Ferreira Minicucci, Juli Thomaz de Souza, Rodrigo Bazan

**Affiliations:** ^1^Medical School, São Paulo State University (UNESP), Botucatu, Brazil; ^2^Department of Physical Therapy, Federal University of Triângulo Mineiro, Uberaba, Brazil; ^3^Department of Neurology, Federal University of Paraná, Curitiba, Brazil

**Keywords:** stroke unit care, cost–benefit analysis, underfunding, benchmark, middle-income countries

## Abstract

**Background:**

Since the implementation of the stroke care line in Brazil, the relationship (adequacy) of costs spent during hospitalization with the Brazilian Ministry of Health indicators for a stroke unit have not yet been analyzed.

**Aims:**

This study aimed to assess the adequacy of a comprehensive stroke center for key performance indicators and analyze the costs involved in hospitalization. We verified the association between stroke severity at admission and care costs during hospitalization.

**Methods:**

A retrospective medical chart review of 451 patients was performed using semiautomatic electronic data from a single comprehensive stroke center in Brazil between July 2018 and January 2020. Clinical and resource utilization data were collected, and the mean acute treatment cost per person was calculated. The Kruskal–Wallis test with Dunn’s post-test was used to compare the total costs between stroke types and reperfusion therapies. A robust linear regression test was used to verify the association between stroke severity at hospital admission and the total hospitalization costs. Good adequacy rates were observed for several indicators.

**Results:**

Data from 451 patients were analyzed. The stroke unit had good adaptation to key performance indicators, but some critical points needed revision and improvement to adapt to the requirements of the Ministry of Health. The average total cost of the patient’s stay was the USD 2,637.3, with the daily hospitalization, procedure, operating room, and materials/medication costs equating to USD 2,011.1, USD 220.7, USD 234.1, and USD 98.8, respectively. There was a positive association between the total cost and length of hospital stay (*p* < 0.001).

**Conclusion:**

The stroke unit complied with most of the main performance indicators proposed by the Brazilian Ministry of Health. Underfunding of the costs involved in the hospitalization of patients was verified, and high costs were associated with the length of stay, stroke severity, and mechanical thrombectomy.

## Introduction

Stroke varies between first and second leading cause of death worldwide and the stroke burden in terms of disability-adjusted life-years is increasing (1). One of the biggest challenges in stroke epidemiology is that most strokes occur in populations of low-to middle-income countries, where health systems are less effective and epidemiological data are scarce (2). Stroke can lead to high health system costs during both hospitalization and post-discharge rehabilitation periods. Currently, approximately 3–4% of the total healthcare expenditure in Western countries is devoted to the follow-up, hospitalization, and rehabilitation of stroke patients (3). Christensen et al. found that the mean initial hospitalization costs for stroke patients in Brazil are substantial and are primarily driven by the intensity of hospital treatment and in-hospital complications (4).

With the expected increase in the incidence of stroke in Brazil in recent decades, the Brazilian Ministry of Health has implemented a stroke care system that includes the implementation of stroke units and regulation of quality indicators for patient monitoring and management (5). The stroke care line includes several healthcare points with predefined flows including popular health education, basic care actions, urgency and emergency services (hospital, fixed, and mobile), stroke and rehabilitation units, outpatient care, and social reintegration (6). Within the stroke care line flow, emphasis should be placed on the importance of emergency stroke care centers (stroke units) because stroke costs are high during hospitalization (4).

Stroke units can be organized according to the stroke care guidelines, and the costs vary due to the heterogeneous healthcare systems prevailing in low- and middle-income countries (7, 8). Stroke care in Brazil has undergone significant changes over the past decade. Several important steps have been implemented and are reflected in the overall better care of stroke patients in Brazil (6). Key performance indicators for stroke care are useful for all hospitals to examine their data and improve their quality of care. However, since the implementation of the stroke care line in Brazil, the relationship (adequacy) of costs spent during hospitalization with the Brazilian Ministry of Health indicators for a stroke unit have not yet been analyzed. In view of the importance of the impact of stroke on Brazilian public health, the present study aimed to assess the adequacy of a comprehensive stroke center (stroke unit level III) for the key performance indicators required by the Brazilian Ministry of Health and to analyze the costs involved in the hospitalization of these patients. In addition, we verified the association between stroke severity at admission and care costs during hospitalization in the stroke unit for patients who experienced ischemic stroke.

## Materials and methods

### Design, setting, and participants

This cross-sectional retrospective study used our stroke database from the electronic medical records of patients hospitalized at the Comprehensive Stroke Center of the Clinical Hospital of Botucatu Medical School, Brazil between July 2018 and January 2020 (9). Patients of both sexes who were hospitalized during the study period and whose data were correctly logged in the database were included. This study was approved by the Institutional Ethics Committee (approval number:4.320.028).

### Data collection

Information was retrieved from the stroke databank created by completing the hospital discharge form in the electronic medical records of patients hospitalized in the stroke unit during the study period. The data collected included sex, age, clinical information, previous use of medications, history of comorbidities prior to stroke, previous modified Rankin scale score, stroke severity according to the National Institute of Health Stroke Scale (NIHSS) score at admission, route of arrival to the service, cerebral reperfusion treatment, examinations performed during hospitalization, and hospital discharge schedule.

### Assessment of key performance indicators

The 13 indicators proposed by Resolution MS/GM No. 665, released by the Ministry of Health on April 12, 2012, which implements the stroke care line for patient monitoring and disease management in all units that treat stroke in Brazil, were evaluated as healthcare measures employed to improve the functioning of the unified health system for patients affected by stroke, in which emergency care centers that qualified as unit types I, II, or III are foreseen (5).

### Cost analysis of hospitalization for stroke

Absorption costing is a method used to calculate the cost of an institution and incorporates all manufacturing costs (materials, labor, and direct, fixed, and variable costs), translating these expenses into unit costs through the apportionment of expenses and allocation to various products and services, so that each center receives what it is entitled to by calculation or attribution (10).

The costs were divided into the patient’s daily rate, which corresponds to expenses related to human resources and services provided to the patient, such as care provided by a multidisciplinary team, cleaning, used trousseau, warehouse, and diet, which are divided in the form of apportionment in an equal way upon admission to the stroke unit; the amount spent on procedures, which includes the money used for the passage of enteral and vesical tubes, venipuncture, electrocardiogram, lumbar puncture, thrombectomy, and any type of procedure performed during hospitalization for stroke; the amount spent on every procedure that requires an operating room which involves a team and specific materials for surgery; the total expenditure on materials and drugs, which includes all material and drugs used in the treatment of the patient during hospitalization; and, finally, the sum of all these expenses was calculated to obtain the average cost of the patients hospitalized for stroke (10).

Indirect costs are paid through hospital apportionment, which implies that areas that do not have their own cost center (laundry, cleaning, equipment maintenance, and information technology) have their expenses divided among all sectors of the hospital according to the size in square meters of the local area, number of employees, and number of appliances and equipment on-site (11). The revenue available for the care of patients in the stroke unit came from the Brazilian public healthcare system to pay all expenses to the patients in accordance with The Management System of Procedures, Medical, Drugs, Orthotics, Prosthetics and Special Materials in Brazil. All procedures and examinations that exceed the amount foreseen within the Brazilian public healthcare system schedule are accounted for as hospital expenses and are charged separately as procedures (12).

The comprehensive care unit for patients with stroke has financial support from the Brazilian government of US$153.44 per day per bed, and this assistance will cover the patient’s stay in the unit for a maximum period of 15 days of hospitalization. For this transfer to occur, the stroke unit must be included in the Regional Action Plan and must carefully follow the requests imposed by the plan (13). In addition to the amount received by the Brazilian Government, the Hospital receives funds from the Health Department and agreements to complement the expenses with patient care. The average values of costs and revenue in Brazilian reals (BRL) were converted to USD based on purchasing power parity in 2019, calculated by the Organization for Economic Cooperation and Development, in which 1 USD = 2.281 BRL (14).

### Statistical analysis

The Kruskal–Wallis test with Dunn’s *post-hoc* test was used to compare the total costs between stroke types (ischemic and hemorrhagic) and between types of reperfusion therapy (thrombolysis, thrombectomy, and conservative treatment). A robust linear regression test adjusted for sex and age was used to verify the association between stroke severity (NIHSS score at hospital admission) and the total hospitalization costs of patients with ischemic stroke. The results were expressed as coefficients and 95% confidence intervals. The significance level was set at 5%. Analyses were performed using Stata/SE v13 software (StataCorp. 2013. Stata Statistical Software: Release 13. College Station, TX, StataCorp LP).

## Results

Data from 451 patients admitted to the stroke unit during the study period were evaluated: 264 (58.5%) patients were male, the mean age was 70 ± 15.8 years, 321 (71.2%) had an ischemic stroke, 34 (7.5%) had a hemorrhagic stroke, the median length of hospital stay was 5 [4–8] days, and the median NIHSS score at admission was 3 [1–8]. Regarding comorbidities, 340 (75.4%) patients had hypertension, 134 (29.7%) had diabetes mellitus, and 114 (25.3%) had dyslipidemia. The previously modified Rankin scale score for 355 patients with ischemic and hemorrhagic stroke was 0 in 208 patients (58.6%), 1 in 68 patients (19.1%), 2 in 30 patients (8.4%), 3 in 29 patients (8.2%), 4 in 18 patients (5.1%), and 5 in 2 patients (0.6%). There were three deaths (8.8%) in patients with hemorrhagic stroke and 23 deaths (7.2%) in patients with ischemic stroke (*p* = 0.995).

A total of 225 (49.9%) patients were from the city of Botucatu and the remaining patients were referred from neighboring cities belonging to the geographical region covered by the clinical hospital. Regarding the arrival route to the hospital, 110 (24.4%) patients visited the mobile emergency care service, 172 (38.1%) were referred to the vacancy center and the Health Offers and Services Regulation Center, 19 (4.2%) had an in-hospital stroke and were transferred from other wards to the stroke unit, 29 (6.4%) came to the service spontaneously, and 121 (26.8%) were referred to the stroke unit from the municipal emergency service.

Table 1 shows the parameters evaluated for each key performance indicator, the target population for each indicator, the number of patients who fit these parameters, and the adequacy (in percentages) of each key performance indicator.

**Table 1:** Key performance indicators for patients admitted to a stroke unit (SU) according to resolution no. 665 for implementing stroke treatment, as published by the Brazilian Ministry of Health, and the percentage of adequacy.

Key performance indicators	Assessed population	Patients that fit the key performance indicators and the percentage of adequacy
Prophylaxis for deep vein thrombosis initiated in the first 48 h after admission	All patients with IS admitted to the SU (n = 321)	290 (90.3%)
Hospital discharge using antiplatelet therapy in patients with NCIS	All patients with NCIS admitted to the SU (n = 273)	239 (87.5%)
Hospital discharge using oral anticoagulation for patients with AF	All patients with IS and AF admitted to the SU (n = 43)	22 (51.2%)
Use of AA, when indicated, started until the second day of hospitalization	All patients with IS admitted to the SU (n = 321)	304 (94.7%)
Hospital discharge using statin for patients with AS	All patients with AS admitted to the SU (n = 74)	71 (95.9%)
Hospital discharge with prophylactic therapy and rehabilitation plan	All patients with IS admitted to the SU (n = 321)	304 (94.7%)
Percentage of patients with stroke admitted to the SU	All patients admitted to the SU (n = 451)	355 (78.7%)
Length of hospital stay for patients with stroke, aiming to reduce it	All patients with stroke admitted to the SU (n = 355)	≤ 3 days (30.0%) 4–6 days (50.0%) 7–8 days (20.0%)
Complications: DVT, pressure injury, pneumonia, and urinary infection	All patients with stroke admitted to the SU (n = 355)	DVT: 2 (0.56%) Pressure injury: 0 (0%) Pneumonia: 32 (9.0%) Urinary infection: 37 (10.4%)
ICD-10 specific type of stroke at hospital discharge	All patients with stroke admitted to the SU (n = 355)	78 (21.9%)
Hospital mortality due to stroke	All patients with stroke admitted to the SU (n = 355)	26 (7.3%)
Door-to-CT scan time < 25 min	All patients admitted to the SU (n = 451)	221 (69.0%)
Door-to-needle time < 60 min	All patients with IS submitted to thrombolytic therapy (n = 38)	22 (67.8%)

SU, stroke unit; IS, ischemic stroke; DVT, deep vein thrombosis; NCIS, non-cardioembolic ischemic stroke; AF, atrial fibrillation; AA, antiplatelet agents; AS, atherothrombotic stroke; CT scan, computed tomography. The results were expressed as numbers and percentages.

Regarding the analysis of the costs of hospitalization in the stroke unit during the study period, the average total cost of the patient’s stay in the stroke unit was USD 2,637.3 (1,625.1–4,092.9), with USD 2,011.1 (1,316.0–3,158.9) spent on daily hospitalization, USD 220.7 (121.7–346.7) spent for procedures, USD 234.1 ± 1,028.2 spent on operating room costs, and USD 98.8 (45.6–217.9) spent on materials and medications. The costs assessed separately by type of cerebrovascular disease are shown in Table 2, and the total costs assessed separately by type of reperfusion therapy are shown in Table 3.

**Table 2:** Hospital total costs separated by stroke type from July 2018 to January 2020 per patients admitted to a Brazilian tertiary stroke unit.

Variable	IS (*n* = 321)	HS (*n* = 34)	TIA (*n* = 36)	*P*
Total costs (USD)	2,806.4 (1,788.1–4,264.5)	3,400.2 (2,040.7–6,880.2)	1,908.2 (1,472.8–3,194.5)	<0.001

*Post-hoc* analysis: Difference between ischemic stroke and transient ischemic attack (*p* < 0.001), and between hemorrhagic stroke and transient ischemic attack (*p* < 0.001). IS, ischemic stroke; HS, hemorrhagic stroke; TIA, transient ischemic attack; USD, American dollar. Kruskal–Wallis test with Dunn’s post-test is performed. Results are expressed as median and 25th and 75th percentiles.

**Table 3:** Hospital total costs of patients with ischemic stroke separated by treatments from July 2018 to January 2020 per patients admitted to a Brazilian tertiary stroke unit.

Variable	IS—Conservative treatment (*n* = 283)	IS—Thrombolysis (*n* = 32)	IS—Thrombectomy (*n* = 6)	*P*
Total costs (USD)	2,725.3 (1,758.9–4,037.8)	3,636.9 (2,123.9–6,656.8)	11,657.2 (7,449.1–16,843.6)	<0.001

*Post-hoc* analysis: Difference between thrombectomy and thrombolysis (*p* < 0.001), and between thrombectomy and conservative treatment (*p* < 0.001). IS, ischemic stroke; USD, American dollar. Kruskal–Wallis test with Dunn’s post-test is performed. Results are expressed as median and 25th and 75th percentiles.

**Figure 1:**
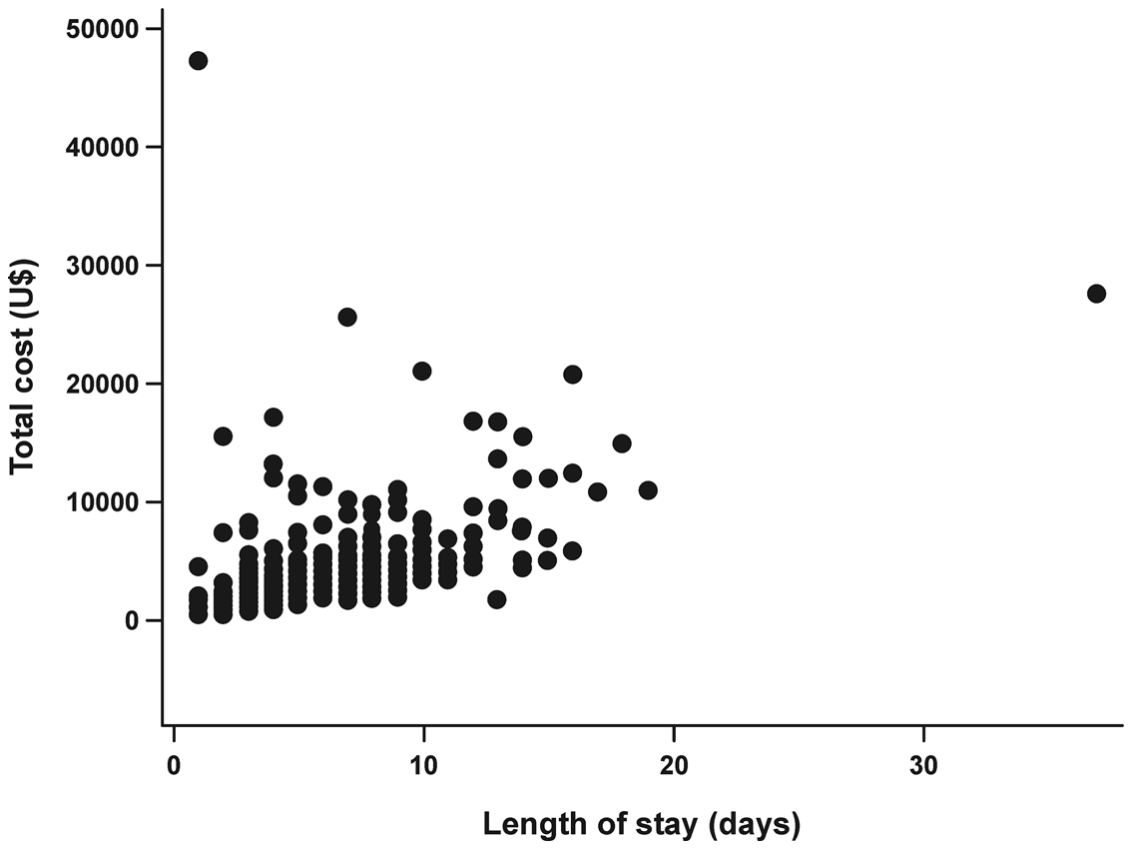
Association between length of stay (days) and total cost (USD).

An association was found between stroke severity and hospitalization costs in patients who experienced ischemic stroke during the study period. When evaluating the association without adjusting for sex and age, it was found that for every 1-point increase in the NIHSS scores, the cost increased by USD 154.6 (47.3–261.9; *p* = 0.005; Table 4); when we adjusted the equation for sex and age, the cost increased by USD 161.5 (50.1–272.8; *p* = 0.005) for each 1-point increase in the NIHSS score (Table 5). In addition, there was a positive association between the total cost and length of hospital stay (r = 0.734; *p* < 0.001; Figure 1).

**Table 4:** Robust linear regression for prediction of hospital cost.

Variable	Beta	CI95%	*P*
NIHSS	154.6	47.3 to 261.9	0.005

NIHSS, National Institute of Health Stroke Scale.

**Table 5:** Robust linear regression for prediction of hospital cost adjusted by age and sex.

Variable	Beta	CI95%	*P*
Age	−24.3	−53.4 to 4.9	0.103
Male	578.7	−221.1 to 1378.4	0.156
NIHSS	161.5	50.1 to 272.8	0.005

NIHSS, National Institute of Health Stroke Scale.

## Discussion

This study highlights the challenges in adapting a stroke unit to the Brazilian Ministry of Health framework and warns about the costs and underfunding of a stroke unit in Brazil. Overall, the stroke unit had good adaptation to the key performance indicators, but some critical areas required revision and improvement to adapt to the requirements of the Brazilian Ministry of Health. In addition, the longer length of hospital stays, more severe stroke, and patients who underwent mechanical thrombectomy were more expensive to treat and monitor than those who received only thrombolysis or conservative treatment. However, positive results were found regarding stroke care, such as high rates of suitability for indication of antiplatelet therapy in patients with noncardioembolic ischemic stroke and use of statins for atherothrombotic stroke at hospital discharge, secondary prophylaxis plans and rehabilitation, indication of antiplatelet use from the second day of hospitalization, short hospital stay, and low mortality rates compared with other stroke care centers (5).

In our study, 51.2% of patients with atrial fibrillation (AF) were discharged from the hospital with a prescription for oral anticoagulants. Individuals who have experienced a stroke or AF have a direct indication for the use of anticoagulants; however, this indication must be evaluated on an individual basis (15). Patients with minor strokes and transient ischemic attack (TIA) who have AF have the possibility of being discharged from the hospital with an indication for oral anticoagulants, while individuals with more severe strokes have a greater risk of bleeding when anticoagulation therapy is initiated early; therefore, it is recommended to start anticoagulants 14 days post stroke. Consequently, in most cases, this indication occurs only after hospital discharge (15). The risk of bleeding, socioeconomic issues that involve the patient, prognosis after stroke, frailty, and risk of falls, in addition to the difficulties of adherence to treatment, are observed during the process of indication of an anticoagulant and are considered at the time of prescription (16).

Regarding post-stroke complications in our sample, the low rates of pneumonia and deep vein thrombosis (DVT) and the absence of pressure injuries should be highlighted. These complications have a significant impact on morbidity and mortality, length of hospital stay, and increased care costs. Pneumonia is a major concern, as it can prolong hospital stay and increase the risk of mortality. Immobility, presence of oropharyngeal dysphagia, use of a nasoenteral tube, and stroke severity are directly linked to the onset of pneumonia and its prognosis (17, 18). Despite the intense multidisciplinary care provided in stroke units, pneumonia still frequently occurs, with incidence rates ranging from 6 to 14% in this population (19–21). The low rate and occurrence of DVT can be explained by the importance of prophylaxis with the use of antiplatelet agents, hydration, and strategies of early mobilization; similarly, the absence of pressure injuries may be the result of patients being under constant surveillance by a highly skilled multidisciplinary team (22–27).

Although our results show good adequacy rates for several indicators, some points need to be reviewed and improved. For example, the low rate of hospital discharges with the specific International Classification of Diseases 10th Revision (ICD-10) denomination may indicate the need for better guidance to assist physicians as every patient admitted to the stroke unit undergoes a complete investigation to determine the type of stroke and etiology for better decision-making during in-hospital treatment and adequate planning for post-discharge. Therefore, not completing the ICD-10 correctly can mask the quality of the service received at the center and give the impression that investigational examinations are not performed. To better adapt this indicator, it is necessary to continue providing educational training to attending physicians, demonstrating the importance of correctly filling in this information, in addition to improving the adequacy of the electronic medical record system to avoid this kind of problem.

It was also highlighted that only a small number of patients who underwent thrombolytic treatments had a door-to-CT scan time < 25 min and door-to-needle time < 60 min. These findings could be related to the difficulties in the assistance flowchart for these patients, which may have an impact on suitability rates. Thus, indications and suitability rates for thrombolytic treatments may be overlooked and underestimated. To better adapt to these indicators, the assistance flowchart needs to be integrated into all stroke survival chains, including mobile emergency support units, general population knowledge about the warning signs of stroke, integration with other areas of the medical clinic, and continuing education of assistant teams in the emergency room and radiology services. All care measures must be planned and managed by an autonomous stroke unit with periodic training to optimize these indicators. However, for this management to be efficient, annual budget corrections are necessary to fully finance the actions of the stroke unit.

This study also found that the costs for patients staying in the stroke unit were similar to those reported in the literature (4, 28). However, costs were higher in patients undergoing cerebral reperfusion therapy, especially in those who underwent mechanical thrombectomy. However, a recent study in a Brazilian population showed that although costs associated with this procedure are high, there are long-term benefits related to the procedure, such as increased life expectancy and reduction of future costs to the public health sector (29). It was also observed that the more severe the ischemic stroke, regardless of sex and age, the higher the expenditure in the stroke unit. This association can be attributed to the greater need for care, number of associated comorbidities, and longer hospital stay (30). Finally, regarding stroke type, the results showed that patients with intraparenchymal hemorrhage do not cost more to monitor and manage than those with ischemic stroke because, most of the time, they are referred and treated in an intensive care unit. In contrast, the low costs for patients with TIA could justify the transfer of these individuals to a specific outpatient clinic or even to a reserved bed within the unit to avoid unnecessary expenses.

The costs involved in the care of patients hospitalized in the Stroke Unit are high, and it is difficult to maintain the quality of this care. Based on the economic model adopted at the institution, it can be said that the Brazilian Ministry of Health is underfunding these costs, which cover only a small portion of the expenses, and the rest is the responsibility of the hospital itself. Our data show that only approximately 6% of the costs are covered by the Unified Health System, which leads us to believe that there is indeed an underfunding by the Brazilian government to maintain the quality of care for stroke patients. To maintain the adequacy of stroke units according to the prerequisites of the Ministry of Health, it is necessary to review this financing with the government so that more funds are made available to hire qualified human practitioners and adequate structures for the diagnosis, treatment, and rehabilitation of stroke to enhance the recovery of affected individuals. One suggestion is implementing a tripartite funding-sharing system in which the federal, state, and municipal governments participate in funding this service, which would significantly impact the entire region served by the hospital.

The major limitation of this study is that the stroke unit did not have a management model with autonomy for economic management and the calculation of costs and collections. The unit depends on the central economic management of the hospital, which can mask or make it difficult to obtain real data on costs per hospitalization. Finally, verifying the strengths and weaknesses of stroke care allows for a better visualization of what should be continued and what can be improved regarding care for these patients. Our results indicate that the care provided in this specific unit and the adequacy of the indicators can help prevent secondary complications, improve prognosis, reduce the length of hospital stay, and consequently, reduce the costs of stroke, enabling an improvement in the quality of healthcare assistance in the coming years.

## Conclusion

The stroke unit assessed in this study complied with most of the main performance indicators proposed by the Brazilian Ministry of Health, except for some critical points that require improvement, showing the need for constant patient surveillance and continuing education for incoming and pre-existing staff to adapt to the evolving proposed guidelines for stroke care to improve patient survival and prognosis. Underfunding of costs involved in the hospitalization of patients was verified, and high costs were associated with the length of stay, stroke severity, and mechanical thrombectomy.

## Data availability statement

The raw data supporting the conclusions of this article will be made available by the authors, without undue reservation.

## Ethics statement

The studies involving humans were approved by Institutional Ethics Committee (approval number: 4.320.028). The studies were conducted in accordance with the local legislation and institutional requirements. The participants provided their written informed consent to participate in this study.

## Author contributions

NF: Methodology, Writing – original draft, Writing – review & editing, Investigation, Project administration. GL: Methodology, Writing – original draft, Writing – review & editing, Supervision. SB: Methodology, Resources, Supervision, Validation, Writing – review & editing. LM: Methodology, Visualization, Writing – original draft. FW: Methodology, Project administration, Visualization, Writing – review & editing. DS: Supervision, Visualization, Resources, Writing – review & editing. CC: Funding acquisition, Project administration, Resources, Writing – review & editing. GM: Methodology, Investigation, Project administration, Resources, Supervision, Writing – review & editing. CF: Conceptualization, Investigation, Project administration, Resources, Supervision, Writing – review & editing. PB: Resources, Supervision, Writing – review & editing, Funding acquisition, Project administration. ML: Methodology, Writing – review & editing, Visualization. MM: Methodology, Supervision, Writing – review & editing, Data curation, Validation. JS: Investigation, Methodology, Project administration, Supervision, Writing – review & editing, Visualization, Writing – original draft. RB: Conceptualization, Funding acquisition, Investigation, Methodology, Project administration, Resources, Supervision, Writing – review & editing.
